# FK506 and Cyclosporin A Enhance IL-6 Production in Monocytes: A single-Cell Assay

**DOI:** 10.1155/S0962935194000529

**Published:** 1994

**Authors:** K. Murayama, M. Sawamura, H. Murakami, J. Tamura, T. Naruse, J. Tsuchiya

**Affiliations:** 1 Third Department of Internal Medicine Gunma University School of Medicine Japan; 2 Division of Blood Transfusion Gunma University School of Medicine Japan; 3 College of Medical Care and Technology Gunma University Gunma Maebashi Japan

## Abstract

The effect of FK506 and cyclosporin A (CsA) on the production of interleukin 6 (IL-6) in adherent monocytes was studied at a single-cell level by the avidinbiotin- peroxidase complex methods. The percentage of IL-6-producing monocytes increased when stimulated with lipopolysaccharide (LPS) at concentrations between 10 ng/ml and 10 μg/ml, in a dose dependent manner. Both FK506 and CsA enhanced the percentage of IL-6- producing monocytes stimulated with 100 pg/ml-1 μg/ml of LPS up to values near those obtained with 10 μg/ml of LPS. The enhancement by FK506 and CsA was not seen when monocytes were stimulated with a high concentration of LPS (10 μg/ml). When monocytes were stimulated with a low concentration of LPS (10 ng/ml), FK506 and CsA enhanced IL-6 production in a dose dependent manner, at a drug concentration of 0.12 nM–1.2 μM (0.1–1 000 ng/ml) for FK506 and 0.83 nM–8.3 μM (1–10 000 ng/ml) for CsA. The optimal effect of FK506 was achieved at a concentration 7-fold lower than that of CsA. In contrast, production of turnout necrosis factor-α (TNFα and interleukin 1β (IL-1β) was slightly suppressed by FK506 and CsA at the concentrations tested. Moreover, pretreatment of monocytes with FK506 and CsA had a significant enhancing effect on LPS-induced IL-6 production, while treatment with FK506 or CsA after LPS stimulation had no effects on IL-6 production, suggesting that the enhancing effect of each drug is exerted before LPS stimulation or at an early stage of the post-receptor pathway after LPS stimulation. These experiments demonstrate that FK506 and CsA can selectively enhance IL-6 production in monocytes under certain conditions *in vitro* and, possibly, also *in vivo*.


Mediators of Inflammation 3, 375–380 (1994)

### FK506 and cyclosporin A enhance IL-6 production in monocytes: a single-cell assay

K. Murayama,[1] M. Sawamura,[1,2,CA] H. Murakami,[1,2] J. Tamura,[1] T. Naruse[1,2] and J. Tsuchiya[3]

[1]Third Department of Internal Medicine, [2]Division of Blood Transfusion, Gunma University School of Medicine, and [3]College of Medical Care and Technology, Gunma University, Maebashi, Gunma, Japan

[CA] Corresponding Author

**The effect of FK506 and cyclosporin A (CsA) on the production of interleukin 6 (IL-6) in adherent monocytes was studied at a single-cell level by the avidin–biotin–peroxidase complex methods. The percentage of IL-6-producing monocytes increased when stimulated with lipopolysaccharide (LPS) at concentrations between 10 ng/ml and 10 μg/ml, in a dose dependent manner. Both FK506 and CsA enhanced the percentage of IL-6-producing monocytes stimulated with 100 pg/ml–1 μg/ml of LPS up to values near those obtained with 10 μg/ml of LPS. The enhancement by FK506 and CsA was not seen when monocytes were stimulated with a high concentration of LPS (10 μg/ml). When monocytes were stimulated with a low concentration of LPS (10 ng/ml), FK506 and CsA enhanced IL-6 production in a dose dependent manner, at a drug concentration of 0.12 nM–1.2 μM (0.1–1 000 ng/ml) for FK506 and 0.83 nM–8.3 μM (1–10 000 ng/ml) for CsA. The optimal effect of FK506 was achieved at a concentration 7-fold lower than that of CsA. In contrast, production of tumour necrosis factor-α (TNFα) and interleukin 1β (IL-1β) was slightly suppressed by FK506 and CsA at the concentrations tested. Moreover, pretreatment of monocytes with FK506 and CsA had a significant enhancing effect on LPS-induced IL-6 production, while treatment with FK506 or CsA after LPS stimulation had no effects on IL-6 production, suggesting that the enhancing effect of each drug is exerted before LPS stimulation or at an early stage of the post-receptor pathway after LPS stimulation. These experiments demonstrate that FK506 and CsA can selectively enhance IL-6 production in monocytes under certain conditions *in vitro* and, possibly, also *in vivo*.**

**Key words:** IL-1β, IL-6, Immunosuppressant, Lipopolysaccharide, TNFα

### Introduction

Cyclosporin A (CsA) is a representative immunosuppressive drug, effective in the treatment of haematologic disorders such as aplastic anaemia and pure red cell aplasia.[1] FK506, a macrolide antibiotic obtained from *Streptomyces tsukubaensis*,[2] has a powerful and selective anti-T cell effect, similar to that of CsA.[3–6] Moreover, some biological roles for the two agents, distinct from T-cell immunosuppression, have been defined.[7–9] However, their effects on human monocytes are still unclear. Monocytes can produce several cytokines such as interleukin 6 (IL-6),[10] tumour necrosis factor-α (TNFα) and interleukin 1β (IL-1β). Andersson *et al.*[11] reported these two drugs had no effect on IL-6 production in monocytes after LPS stimulation.

The present study examined whether FK506 and CsA would influence the production of IL-6 in monocytes at a single-cell level by the avidin–biotin–peroxidase complex (ABC) methods. It is shown here that FK506 and CsA selectively enhance the number of IL-6 producing monocytes when stimulated with low concentrations of LPS, although neither of them had an enhancing effect when monocytes were stimulated with a high concentration of LPS.

### Materials and Methods

*Immunosuppressants:* FK506 (molecular weight (MW) 1202.63) was kindly provided by Fijisawa Pharmaceutical Co. Ltd (Osaka, Japan). Cyclosporin A (CsA) (MW 822.05) was purchased from Sandoz, Ltd (Tokyo, Japan). FK506 was diluted in 100% ethanol to a concentration of 1.2 mM (1 mg/ml) and further diluted with RPMI 1640 culture medium to obtain working concentrations. CsA was diluted with RPMI 1640. Both drugs were used without preservatives and were freshly prepared for each experiment.

*Preparation of multiple-square slides:* Multiple-square slides with 3 × 7 squares (4 mm × 4 mm) were made on glass slides by drawing straight paral-

© 1994 Rapid Communications of Oxford Ltd lel vertical and horizontal lines with a PAP Pen (Daido Sangyo Co., Ltd, Japan). Hydrophobic lines prevent aqueous solutions in separate wells from leaking out.

*Monocyte separation and cytokine induction:* Mononuclear cells (MNC) were isolated from freshly drawn blood from healthy blood donors by centrifugation on a density gradient (Lymphocyte separation medium, Organon Teknika Corporation, Durham, NC). Cells ($2 \times 10^6$ cells/ml) were then suspended in RPMI 1640 medium supplemented with endotoxin free 5% foetal bovine serum (FCS), 2 mM L-glutamine, 100 U/ml penicillin and 100 μg/ml streptomycin. Ten μl of the cell suspension was placed in each well of multiple-square slides and allowed to adhere for 30 min at 37°C in a humidified atmosphere containing 5% $CO_2$. After incubation, the non-adherent cells were removed by washing with PBS. The plating efficiency of the monocytes was more than 90%. The monocyte purity was more than 98% as assessed by morphology, peroxidase and non-specific esterase staining. This culture medium was endotoxin free as determined by the *Limulus* amoebocyte lysate assay (less than 0.2 ng/ml of endotoxin). Monocyte monolayers were overlaid with fresh culture medium (1% FCS–RPMI 1640) and cultured in the presence of 10 ng/ml of lipopolysaccharide (LPS) from *Escherichia coli* serotype 026:B6 (Sigma Chemical Co, St Louis, MO) for 4 h. After culture, the cells were immediately fixed for detection of cytokine-producing cells by ABC method. In some experiments, the monocytes were cultured with various concentrations of LPS.

*Dose–response curves for FK506 and CsA:* FK506 was used at 10-fold dilutions in each experiment from culture concentrations of 12 pM–1.2 μM (0.01–1 000 ng/ml). CsA was used at concentrations of 83 pM to 8.3 μM (0.1 to 10 000 ng/ml). The two drugs were added from the beginning of the cultures with or without LPS and were present throughout the culture period. LPS-activated monocytes, without FK506 or CsA, served as positive controls. The cells were fixed to detect cytokine-producing cells 4 h after the initiation of LPS stimulation.

*Pretreatment and post-treatment by FK506 or CsA:* Monocytes were incubated with or without 12 nM (10 ng/ml) FK506 or 0.83 μM (1 000 ng/ml) cyclosporin A for 1 h before LPS stimulation. After washing the monocytes with PBS, 10 ng/ml LPS was added to part of the cultures and these were incubated for various periods of time. After washing the monocytes with PBS, 10 μl of 1% FCS–RPMI 1640 was added to the wells and culture was continued. In some experiments, monocytes were stimulated with 10 ng/ml LPS for 30 min washed with PBS and then incubated with or without 12 nM (10 ng/ml) FK506 or 0.83 μM (1 000 ng/ml) CsA for 1 h or 3.5 for both. After washing them with PBS, 10 μl of 1% FCS–RPMI 1640 was added to the wells and culture was continued. The cells on multiple-square slides were fixed to detect cytokine-producing cells 4 h after the initiation of LPS stimulation.

*Immunocytochemical staining of cytokines:* Enzyme immunocytochemistry was performed essentially as previously described to detect cytoplasmic cytokines by an indirect immunofluorescence technique.[12] The cells fixed in phosphate buffered 4% paraformaldehyde were stained using the ABC method. They were then incubated for 30 min with cytokine specific mAb diluted in 0.1% saponin at room temperature. The following mAb were used at a final concentration of 2 μg/ml: anti-IL-6 mAb (mouse IgG1, Genzyme Co., Boston, MA); anti-TNFα mAb (Clone 195, mouse IgG3, Boehringer Mannheim GmbH, Mannheim, Germany); and anti-IL-1β (mouse IgG1, Genzyme Co.). After washing the mAb three times with phosphate buffered saline (PBS) containing 1% Tween 20 and 0.1% saponin, they were incubated with biotin conjugated horse anti-mouse IgG sera diluted in 0.1% saponin–PBS for 20 min. Heat-inactivated human AB serum (5%) was used in this step to reduce nonspecific protein interaction. In the subsequent procedures Vestastain alkaline phosphatase ABC reagents (Vector Laboratories Inc., Burlingame, CA) were used. The specimens were developed in substrate containing 1 mmol/l levamisole to block endogenous enzymes. The specimens were not counterstained. Control cells for each experiment were stained with a myeloma protein of identical subclass (IgG1 and IgG3) at a final concentration of 2 μg/ml. The results are given as the percentage of positively stained cells. This was done by counting more than 500 cells. Unstimulated cells were used as negative controls in each experiment. The S.D. within a multiple-square slide or among different slides was less than 10% of the mean value.

*Statistical evaluation:* The results are expressed as the mean values ± S.D. The statistical significance of differences was determined by Student's *t*-test.

### Results

*Effect of FK506 or CsA on LPS-induced cytokine production in monocytes:* The effect of FK506 and cyclosporin A on cytokine production was studied using an immunocytochemical technique at a single-cell level. Monocytes were cultured with 10 ng/ml LPS for 4 h. With this technique, a local perinuclear cytoplasmic staining pattern was seen in IL-6 producing monocytes (Fig. 1A) as well as TNFα-producing monocytes, while IL-1β-producing

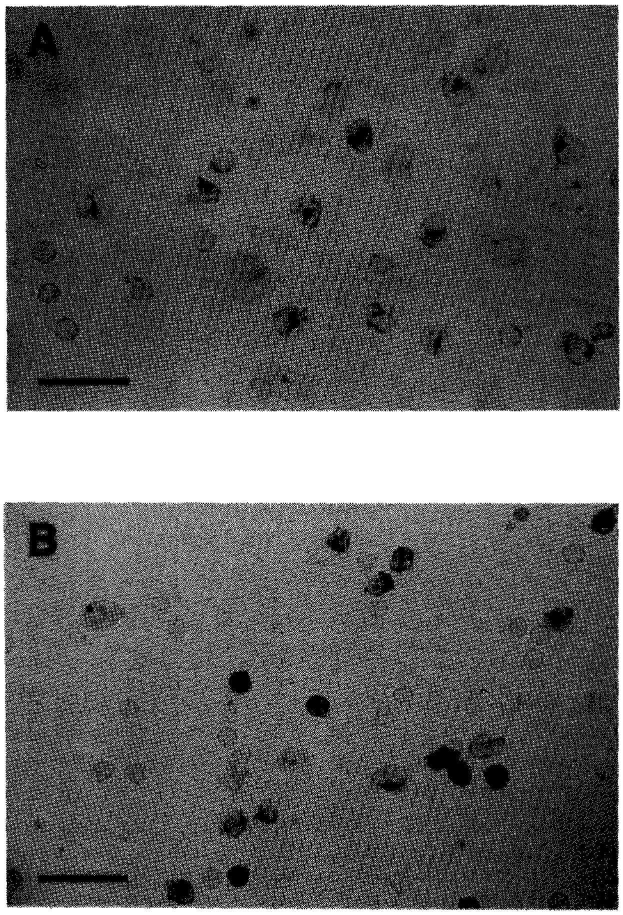

FIG. 1. Immunocytochemical staining of IL-6-producing monocytes 4 h after initiation of culture with LPS (10 ng/ml). (A) IL-6-producing monocytes. (B) IL-1β-producing monocytes. Bar, 30 μm.

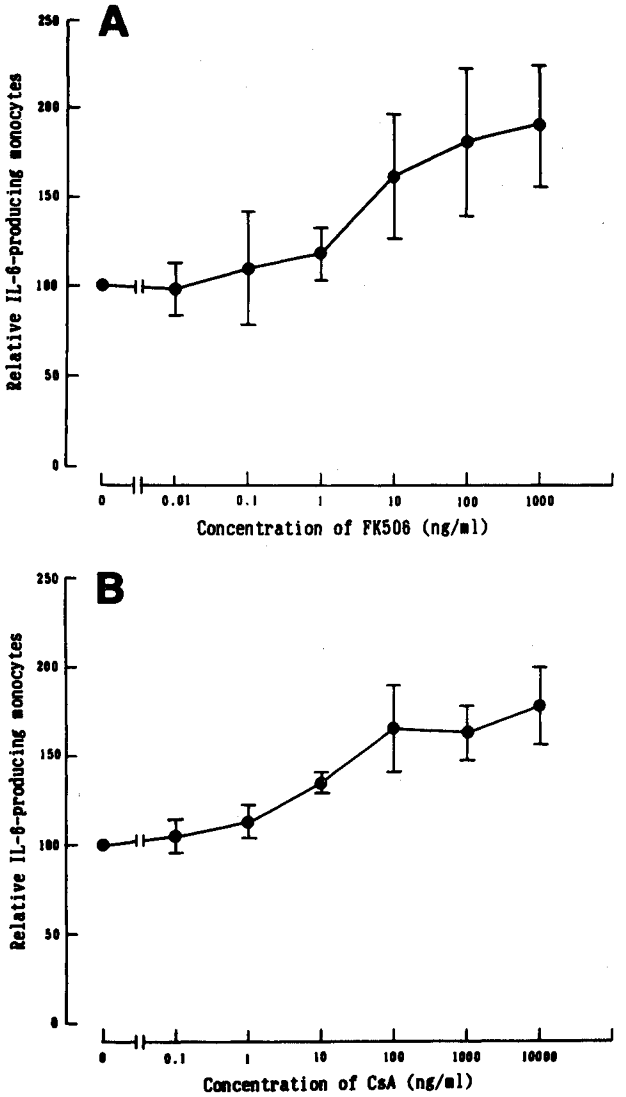

FIG. 2. Dose dependent effects of FK506 or cyclosporin A on IL-6 production by monocytes after LPS stimulation. Monocytes from five donors were cultured with LPS (10 ng/ml) and the indicated concentrations of FK506 (A) or cyclosporin A (B) for 4 h. Data are the mean ± S.D. of five experiments. The effects are expressed as percentage of IL-6-producing monocytes compared with those stimulated with LPS alone.

monocytes showed a diffuse cytoplasmic staining pattern (Fig. 1B).

While cytokine production was rarely seen in the absence of LPS (< 2% monocytes), *in vitro* stimulation with LPS resulted in a prompt increase of IL-6, TNFα and IL-1β production and the maximal number of these cytokine producing monocytes was noted after 4 h of LPS stimulation (data not shown). Approximately 40% of the monocytes produced TNFα and IL-1β within 4 h of LPS stimulation.

The percentage of IL-6-producing monocytes in 4-h cultures from different healthy donors varied widely (range: 14–37%). The response to the two drugs and percentage of IL-6 producing monocytes among different donors also varied. However, both FK506 and CsA increased the percentage of IL-6-producing monocytes in a dose dependent manner (Fig. 2). The increase of IL-6 producing monocytes was observed at drug concentrations between 0.12 nM and 1.2 μM (0.1 and 1 000 ng/ml) for FK506 (Fig. 2A) and between 0.83 nM and 8.3 μM (1 and 10 000 ng/ml) for CsA (Fig. 2B). The concentration of FK506 required to attain half the maximal stimulation of IL-6 production was 6 nM (5 ng/ml), which was calculated as the mean value of IL-6-producing cells for five healthy donors. In the case of CsA, it was 42 nM (50 ng/ml). By comparison, to obtain the same stimulating effect, the required dose of FK506 was 7-fold lower than that of CsA.

In contrast, the percentage of LPS-stimulated TNFα- and IL-1β-producing monocytes was slightly decreased by FK506 and CsA, at doses up to 1.2 μM (1 000 ng/ml) and 8.3 μM (10 000 ng/ml), respectively (Fig. 3). The percentage of TNFα and IL-1β-producing monocytes was assessed every hour from 1 h to 6 h after LPS stimulation under the same conditions; FK506 and CsA had little or no effect on the production of each cytokine (data not shown). Ethanol, which was used as the solvent, had no effect on the production of these cytokines at a 100-fold dilution. The viability of cells remained at more than 95% as assessed by trypan blue dye uptake under the experimental conditions of this study.

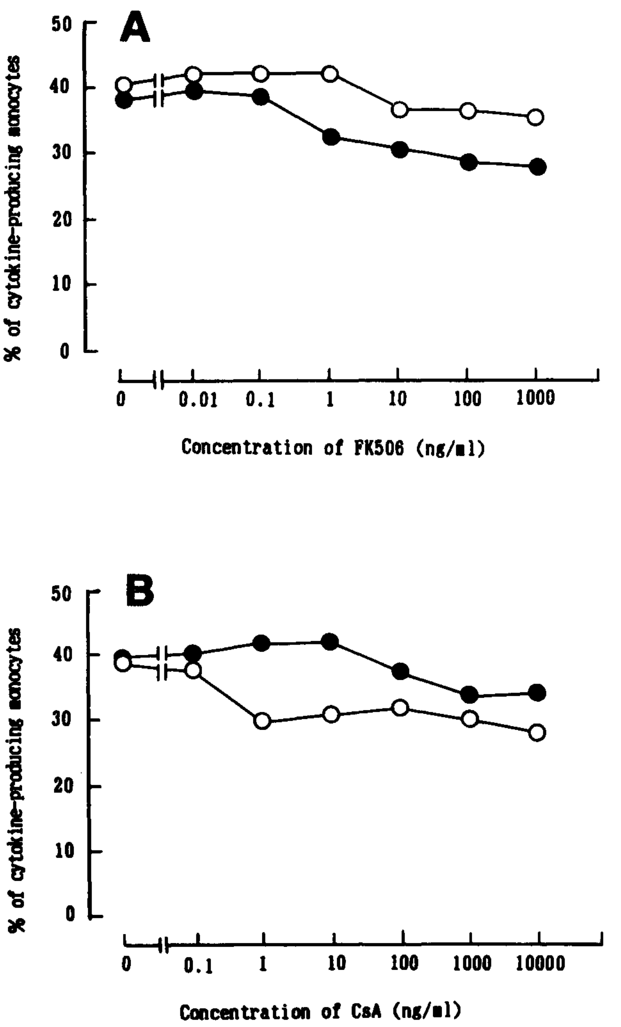

FIG. 3. Effects of FK506 or cyclosporin A on TNFα and IL-1β production by monocytes after LPS stimulation. Monocytes were cultured with LPS (10 ng/ml) and the indicated concentrations of FK506 (A) or cyclosporin A (B) for 4 h. The percentage of TNFα- and IL-1β-producing monocytes was assessed. Representative data from one of three experiments are shown. In the absence of LPS, the percentage of each cytokine-producing monocytes was less than 2% (data not shown). (●) TNFα, (○) IL-1β.

Next, the effect of FK506 and CsA on IL-6-production was examined by stimulating monocytes with various amount of LPS (Fig. 4). Monocytes were cultured with LPS (100 pg/ml–10 μg/ml) and 12 nM (10 ng/ml) FK506, 0.83 μM (1 000 ng/ml) CsA, or medium. After 4 h of culture, the percentage of IL-6-producing cells was assessed. The percentage of IL-6-producing monocytes was about the same (34.6%–36.6%) when stimulated with LPS alone at doses ranging between 100 pg/ml and 10 ng/ml. However, it increased when stimulated with LPS at the concentrations of 10 ng/ml–10 μg/ml, in a dose dependent manner. As shown in Fig. 4, both FK506 and CsA increased the percentage of IL-6-producing monocytes when stimulated with 100 pg/ml–1 μg/ml of LPS. At the concentration of 10 ng/ml of LPS, the percentage of IL-6-producing monocytes cultured with control medium, FK506 and CsA was 36.6%, 67.7% and 63.7%, respectively. That is, a 1.85-fold increase for FK506 and a 1.74-fold increase for CsA

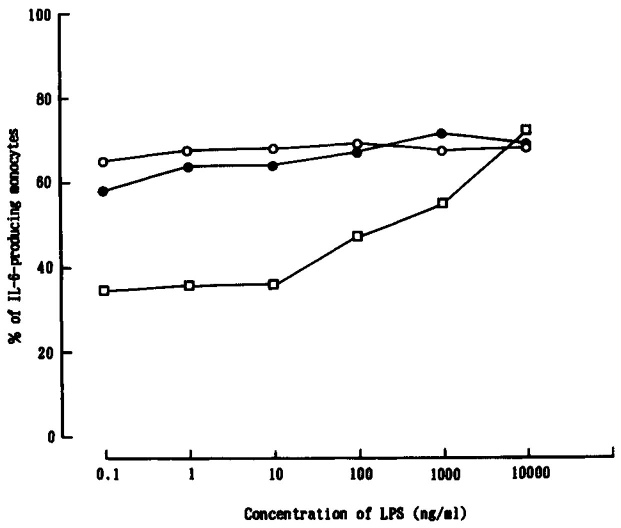

FIG. 4. Effects of FK506 or cyclosporin A on IL-6 production by monocytes stimulated with various concentrations of LPS. Monocytes were cultured with the indicated concentrations of LPS and 12 nM (10 ng/ml) FK506 (○), 0.83 μM (1 000 ng/ml) cyclosporin A (●), or medium (□). After 4 h culture, the percentage of IL-6-producing monocytes was assessed. Representative data from one of four experiments are shown.

was noted. In contrast, when the monocytes were stimulated with 10 μg/ml LPS, the percentage of IL-6-producing monocytes is about the same (72.65% *vs.* 68.2% *vs.* 69.0%); that is, in the presence of a higher concentration of LPS FK506 and CsA did not enhance IL-6 production further.

*Effect of pretreatment or post-treatment of FK506 and cyclosporin A on Il-6 production in LPS stimulated monocytes:* Treatment of monocytes with FK506 or CsA before LPS stimulation increased the percentage of Il-6-producing monocytes after LPS stimulation more than in control cultures (Table 1). Monocytes were incubated with LPS for 5 min, 30 min or 4 h and the percentage of IL-6-producing monocytes was measured 4 h after the addition of LPS. Pretreatment with FK506 increased the percentage of IL-6-producing monocytes to 20.55 ± 0.07%, 22.05 ± 1.20%, and 28.60 ± 2.12%, after 5 min, 30 min and 4 h treatment with LPS, respectively. Pretreatment with CsA also increased the percentage of IL-6-producing monocytes to 22.05 ± 2.05%, 21.85 ± 1.85%, and 25.49 ± 0.49%, respectively. On the other hand, treatment with FK506 and CsA after LPS stimulation had no enhancing effect on the percentage of IL-6-producing monocytes, as shown in Table 1.

### Discussion

In this study it was found that FK506 and CsA selectively enhanced the production of IL-6 in monocytes stimulated with a low concentration of LPS, whereas the percentage of LPS-stimulated TNFα- and IL-1β-producing monocytes was slightly suppressed by FK506 an CsA. In contrast, Andersson *et al.*[9] reported that FK506 and CsA had no effect on LPS induced IL-6 production in monocytes. They

**Table 1.** Effect of cyclosporin A and FK506 on the percentage of IL-6-producing monocytes

| Pretreatment[a] | LPS[b] | Post-treatment[c] | Cytoplasmic IL-6 positive cells (%)[d] |
|---|---|---|---|
| – | 4 h | – | 15.50 ± 1.27 |
| FK506 1 h | 4 h | – | 28.60 ± 2.12[e] |
| CsA 1 h | 4 h | – | 25.49 ± 0.49[e] |
| – | 5 min | – | 12.40 ± 0.87 |
| FK506 1 h | 5 min | – | 20.55 ± 0.07[e] |
| CsA 1 h | 5 min | – | 22.05 ± 2.05[e] |
| – | 30 min | – | 14.15 ± 0.35 |
| FK506 1 h | 30 min | – | 22.05 ± 1.20[e] |
| CsA 1 h | 30 min | – | 21.85 ± 1.85[e] |
| – | 30 min | – | 13.30 ± 0.40 |
| – | 30 min | FK506 1 h | 13.90 ± 0.14 |
| – | 30 min | CsA 1 h | 15.05 ± 0.78 |
| – | 30 min | FK506 3.5 h | 13.75 ± 1.63 |
| – | 30 min | CsA 3.5 h | 14.85 ± 0.07 |
| – | – | – | 0.50 ± 0.50[f] |
| FK506 1 h | – | – | 0.50 ± 0.50[f] |
| CsA 1 h | – | – | 0.50 ± 0.50[f] |

[a]Monocytes were incubated with or without 12 nM (10 ng/ml) FK506 or 0.83 μM (1 000 ng/ml) cyclosporin A for 1 h before LPS stimulation.
[b]Monocytes were stimulated or not stimulated with 10 ng/ml LPS for the indicated periods of time.
[c]Monocytes were incubated with or without 12 nM (10 ng/ml) FK506 or 0.83 μM (1 000 ng/ml) cyclosporin A for 1 h or 3.5 h after LPS stimulation.
[d]Percentages of IL-6-producing cells were determined 4 h after the initiation of LPS stimulation. Data are shown as the mean ± S.D. in triplicate culture.
[e]$p < 0.05$; [f]$p < 0.01$ (*vs.* controls with LPS for 30 min). *p* values are given when statistically significant.

added LPS at a concentration of 100 ng/ml. Our observations suggest that the LPS concentrations employed might account for these differences. Although in our study neither drug had an enhancing effect on the number of IL-6-producing monocytes when stimulated with a high concentration of LPS, both FK506 and CsA did increase the percentage of IL-6-producing monocytes when the cells were stimulated with low concentrations of LPS (10 ng/ml). Secondly, the different procedures might also account for the different results obtained by Andersson *et al.*[9] because multiple-square glass slides were used to separate and culture adherent monocytes as well as for immunocytochemistry.[13,14] On the other hand, it has been suggested that adherence of monocytes would lead to IL-6 production,[15,16] but the present observations did not support these speculations since no or very low IL-6 production (< 2% monocytes) occurred when cells were cultured without LPS.

Stimulating effects by FK506, as observed in this study, are not surprising because it is known that FK506 had a stimulating effect on the growth of colony-forming units/granulocyte–macrophage (CFU–GM) and burst-forming units/erythroid (BFU–E) from peripheral blood and cord blood cells,[9] although the precise mechanism remains unclear.

In the present study, pretreatment with FK506 and CsA followed by LPS increased the percentage of IL-6-producing monocytes but post-treatment with either drug had no effect, suggesting that the enhancing effect of each drug is exerted before LPS stimulation or at an early stage of the post-receptor pathway after LPS stimulation. Therefore, the different effects of FK506 and CsA on monocytes stimulated with low and high concentrations of LPS may be exerted via different receptors. One possible receptor for LPS is CD14, which is the receptor for LPS–LPS binding protein (LBP) on monocytes.[17] In this model, LBP promotes the sensitivity of monocytes to LPS by both lowering the threshold and accelerating the time course of cytokine production. LPS interacts with LBP and the LPS–LBP complex binds to CD14, leading to monocyte activation for cytokine production.[17,18] Monocytes are also stimulated by high concentrations of LPS in the absence of LBP probably via putative LPS receptors that are independent of LBP and CD14.[19] Similarly, in our study, low concentrations of LPS may stimulate monocytes via CD14 but high concentrations of LPS may stimulate monocytes via other LPS receptors on their surface. The different receptors and subsequent intracellular signalling pathways may determine the type of effect of FK506 and CsA. Although various LPS binding sites have been identified on monocytes, no physiological definite LPS receptor has yet been demonstrated.[20] So the precise details of this possibility remain to be determined.

The enhancing effect of FK506 and CsA on monocytes may be due to the inhibition of calcineurin, similar to the T-cell suppressive effect, by the two drugs. FK506 inhibits T-cell function at concentrations 7 to 70 times lower than CsA.[21,22] While FK506 shares no structural similarity with CsA, both drugs appear to inhibit T-cell signalling pathways by similar mechanisms which involve the $Ca^{2+}$–calmodulin dependent serine/threonine phosphatase calcineurin pathway.[23,24] CsA and FK506 inhibit calcineurin after binding to their respective receptor proteins, cyclophilin and FK506-binding protein (FKBP). This inhibition is specific for calcineurin. The CsA–cyclophilin complex and the FK506–FKBP complex were found to bind to and inhibit calcineurin. Accordingly, the observation that the two drugs increased the percentage of IL-6-producing monocyte suggests that inhibition of calcineurin might be related to this enhancement. In the present study the concentration of either FK506 or CsA required for this enhancement is similar to the concentrations at which both drugs can cause T-cell immunosuppression. Moreover, the optimal effect of FK506 was achieved at a concentration 7-fold lower than that of CsA. The concentration of FK506 required to inhibit calcineurin dependent cell function should depend on relative amounts of FKBP and calcineurin in the cells.[25] The concentrations of FKBP and calcineurin in monocytes or lymphocytes are not known, and this makes comparison difficult. Further functional analyses are required to determine the function of calcineurin on monocytes and T cells.

Cytokine production at a single-cell level was studied by an immunocytochemical method performed essentially, as described previously by Sander *et al.* IL-6 and TNFα showed a local, perinuclear staining reflecting the accumulation adjacent to the nucleus, while IL-1β staining has resulted in more diffuse cytoplasmic staining. Similar experiences have been reported by other groups.[12,26] Sander *et al.*[12] mentioned that the characteristic staining pattern of IL-6 and TNFα is seen in cytokine-producing cells rather than during cytokine uptake and it reflects the accumulation of cytokines in the Golgi organelle. The diffuse cytoplasmic staining, on the other hand, would indicate that the intracellular transport of IL-1β follows a different pathway from the classical endoplasmic reticulum–Golgi route.[27] It was confirmed that the ABC method can be used to detect cytoplasmic cytokines. This method is more useful than immunofluorescence techniques because it does not need an immunofluorescence microscope and allowed long-term morphological evaluation.

Dysregulation of IL-6 production is often associated with pathological conditions that involve abnormal B-cell proliferation in multiple myeloma and lymphoproliferative disorders.[10] IL-6 is an essential growth factor for myeloma cells and the elevated serum levels appear to correlate with the severity of the disease.[10] Moreover, IL-6 induces massive plasmacytosis with autoantibody production in IL-6 transgenic mice.[28] On the other hand, CsA-treated patients showed evidence of an increased incidence of polyclonal lymphoproliferative disorders and B cell lymphomas, and their risk of developing lymphoma is related to the degree and duration of their immunosuppression.[29] In addition, CsA could facilitate the development of Epstein–Barr virus (EBV)-transformed B cell lymphomas via potentiation of the IL-6 production.[30] Therefore, the potentiation of the production of IL-6 by FK506 or CsA may have an undesirable outcome on patients with multiple myeloma or lymphoproliferative disorders, where prolonged administration of these drugs might be anticipated.

In conclusion, FK506 and CsA can selectively enhance IL-6 production in monocytes stimulated with low concentrations of LPS, although neither drug can enhance it when monocytes are stimulated with high concentrations of LPS. It should be noted from our observation that the enhancement of cytokine production by FK506 and CsA can occur in the presence of very low amount of LPS and influence the cytokine network *in vitro* and, possibly, also *in vivo*.

### References

1. Leonard EM, Raefsky E, Griffith P, Kimball J, Nienhuis AW, Young NS. Cyclosporine therapy of aplastic anemia, congenital and acquired red cell aplasia. *Brit J Haemat* 1989; **72:** 278–284.
2. Kino T, Hatanaki M, Hashimoto M, *et al.* FK-506, a novel immunosuppressant isolated from a streptomyces. I. Fermentation, isolation, and physicochemical and biological characteristics. *J Antibiot (Tokyo)* 1987: **40:** 1249–1255.
3. Sawada S, Suzuki G, Kawase Y, Takaku F. Novel immunosuppressive agent FK506: *in vitro* effects on the cloned T-cell activation. *J Immunol* 1987; **139:** 1797–1803.
4. Bierer BE, Somers PK, Wandless TJ, Burakoff SJ, Schreiber SL. Probing immunosuppressant action with a nonnatural immunophilin ligand. *Science* 1990; **250:** 556–559.
5. Kimball PM, Kerman RH, Kahan BD. Failure of propylpeptidyl isomerase to mediate cyclosporine suppression of intracellular activation signal generation. *Transplantation* 1991; **51:** 509–513.
6. Tocci MJ, Matokovich DA, Coller KA, *et al.* The immunosuppressant FK506 selectively inhibits expression of early T cell activation genes. *J Immunol* 1989; **143:** 718–726.
7. Arceci RJ, Stieglitz K, Bierer BE. Immunosuppressants FK506 and rapamycin function as reversal agents of the multiple resistance phenotype. *Blood* 1992; **80:** 1528–1536.
8. Hendey B, Klee CB, Maxfield FR. Inhibition of neutrophil chemokinesis on vitronectin by inhibitors of calcineurin. *Science* 1992; **258:** 296–299.
9. Harao A, Kawano Y, Takaue Y. Effects of immunosuppressants, FK506, deoxyspergualin, and cyclosporine A on immature human hematopoiesis. *Blood* 1993; **81:** 1179–1183.
10. Kishimoto T, Hirano T. B-cell stimulatory factor-2 (BSF-2)/interleukin 6 (IL-6). *Annu Rev Immunol* 1988; **6:** 485–512.
11. Andersson J, Nagy S, Groth CG, Andersson U. Effect of FK506 and cyclosporin A on cytokine production studied *in vitro* at a single-cell level. *Immunology* 1992; **75:** 135–142.
12. Sander B, Andersson J, Andersson U. Assessment of cytokines by immunofluorescence and the paraformaldehyde–saponin procedure. *Immunol Rev* 1991; **119:** 65–93.
13. Ziegler-Heitbrock HWL, Strobel M, Kieper D, *et al.* Differential expression of cytokine in human blood monocytes subpopulations. *Blood* 1991; **79:** 503–511.
14. Hamilton JA. Colony stimulating factors, cytokines and monocyte–macrophages — some controversies. *Immunol Today* 1993; **14:** 18–24.
15. Vaquero CJ, Sanceau J, Weissenbach J, Beranger F, Falcoff R. Regulation of human γ-interferon and β-interferon gene expression in PHA-activated lymphocytes. *J Interferon Res* 1986; **6:** 161–170.
16. Horii Y, Muraguchi A, Suematsu S, *et al.* Regulation of BSF-2/IL-6 production by human mononuclear cells. Macrophage-dependent synthesis of BSF-2/IL-6 by T cells. *J Immunol* 1988; **141:** 1529–1535.
17. Wright SD, Ramos RA, Tobias PS, Ulevitch RJ, Mathison JC. CD14, a receptor for complexes of lipopolysaccharide (LPS) and LPS binding protein. *Science* 1990; **249:** 1431–1433.
18. Mathison JC, Tobias PS, Wolfson E, Ulevitch RJ. Plasma lipopolysaccharide (LPS)-binding protein. A key component in macrophage recognition of Gram-negative LPS. *J Immunol* 1992; **149:** 200–206.
19. Schumann RR, Leong SR, Flaggs GW, *et al.* Structure and function of lipopolysaccharide binding protein. *Science* 1990; **249:** 1429–1431.
20. Lynn WA, Golenbock DT. Lipopolysaccharide antagonists. *Immunol Today* 1992; **13:** 271–276.
21. Kino T, Hatanaka H, Miyata S, *et al.* FK506, a novel immunosuppressant isolated from streptomyces. II. Immunosuppressive effect of FK506 *in vitro*. *J Antibiot (Tokyo)* 1987; **40:** 1256–1265.
22. Schreiber SL. Chemistry and biology of the immunophilins and their immunosuppressive ligands. *Science* 1991; **251:** 283–287.
23. Friedman J, Weissman I. Two cytoplasmic candidates for immunophilin action are revealed by affinity for a new cyclophilin: one in the presence and one in the absence of CsA. *Cell* 1991; **66:** 799–806.
24. Liu J, Farmer JD Jr, Lane WS, Friedman J, Weissman I, Schreiber SL Calcineurin is a common target of cyclophilin–cyclosporin A and FKBP–FK506 complexes. *Cell* 1991; **66:** 807–815.
25. Liu J, Albers MW, Wandless TJ, *et al.* Inhibition of T cell signalling by immunophilin-ligand complexes correlates with loss of calcineurin phosphatase activity. *Biochemistry* 1992; **31:** 3896–3901.
26. Hofsli E, Bakke O, Nonstad U, Espevik T. A flow cytometric and immunofluorescence microscopic study of tumor necrosis factor production and localization in human monocytes. *Cell Immunol* 1989; **122:** 405–415.
27. Rubartelli A, Cozzolino F, Talio M, Sitia R. A novel secretory pathway for interleukin 1 beta, a protein lacking a signal sequence. *EMBO J* 1990; **9:** 1503–1510.
28. Suematsu S, Matsuda T, Aozasa K, *et al.* $IgG_1$ plasmacytosis in interleukin 6 transgenic mice. *Proc Natl Acad Sci USA* 1989; **86:** 7547–7551.
29. Cockburn IT, Krupp P. The risk of neoplasms in patients treated with cyclosporine A. *J Autoimmun* 1989; **2:** 723–731.
30. Walz G, Zanker B, Melton LB, *et al.* Possible association of the immunosuppressive and B cell lymphoma-promoting properties of cyclosporine. *Transplantation* 1990; **49:** 191–194.

**Received 15 April 1994; accepted in revised form 8 June 1994**
